# Early nonreactivity in the conjunctival provocation test predicts beneficial outcome of sublingual immunotherapy

**DOI:** 10.1186/s13601-018-0214-y

**Published:** 2018-07-04

**Authors:** Janina Köther, Alicia Mandl, Silke Allekotte, Anatoli Astvatsatourov, Janin Chwieralski, Jan-Patrick Liedtke, Ursula Pieper-Fürst, Esther Raskopf, Kija Shah-Hosseini, Ralph Mösges

**Affiliations:** 1 Institute of Medical Statistics and Computational Biology, Faculty of Medicine, University of Cologne, Cologne, Germany; 2 CRI – Clinical Research International Ltd., Genter Str. 7, 50672 Cologne, Germany; 3 Clinical Trials Centre Cologne, Faculty of Medicine, University of Cologne, Cologne, Germany

**Keywords:** Conjunctival provocation test, Conjunctival allergen challenge, Early responder, Sublingual immunotherapy

## Abstract

**Background:**

Clinical practice needs a common parameter that can provide an early, reliable estimation of the outcome of sublingual immunotherapy (SLIT) in an upcoming pollen season. We investigated whether the conjunctival provocation test (CPT) can predict the beneficial outcome of SLIT in patients with allergic rhinoconjunctivitis after 4 weeks of treatment.

**Methods:**

We conducted two separate prospective, randomized, double-blind, placebo-controlled, multicenter trials. Adults 18–75 years of age received placebo or SLIT tablets containing tree or grass pollen allergoids and underwent CPTs. Participants receiving SLIT were divided into two groups (reactive, nonreactive) according to their CPT reactions after 4 weeks of treatment. These two groups were compared with regard to clinical outcome parameters (total combined score, rhinoconjunctivitis total symptom score, total rescue medication score, well days) assessed during the pollen season for the 14-day (tree) or 30-day (tree/grass) peaks and for the entire 60-day seasons. Participants’ global evaluations of therapy after completing treatment were also compared.

**Results:**

The tree pollen trial randomized 188 participants; 182 participants were evaluable, 76 of whom received SLIT and were suitable for this post hoc analysis. The grass pollen trial included 90 participants; 82 participants were evaluable, 44 of whom underwent SLIT. Comparing SLIT participants who reacted to the CPT after 4 weeks (tree: 77.6%; grass: 79.5%) with those who ceased to show a reaction (tree: 22.4%; grass: 20.5%) (tree: *P *= 0.0001; grass: *P *= 0.003), the total combined score for the 14-day (*P *= 0.017) and 30-day peaks (*P *= 0.042) as well as the rhinoconjunctivitis total symptom score assessed for the 14-day peak (*P *= 0.024) were significantly lower in the nonreactive group of the tree pollen trial. In the grass pollen trial, the nonreactive group rated their SLIT treatment significantly better (*P *= 0.019).

**Conclusions:**

Using clinically meaningful outcome parameters during the pollen season, both trials independently led to similar results when comparing participants’ reactions to the CPT 4 weeks after beginning SLIT. These results suggest that CPT allows an early estimation of allergic rhinoconjunctivitis symptoms before an upcoming season. Thus, the CPT can be used as a valuable parameter to predict the beneficial outcome of ongoing SLIT.

**Trial registration:**

Both trials registered with the Medical Ethics Committee of the North Rhine Medical Council (EudraCT numbers 2012-004916-79 (grass pollen trial) and 2013-002129-43 (tree pollen trial)) and the German Federal Ministry of Health (Paul-Ehrlich-Institut).

## Background

The efficacy and safety of sublingual immunotherapy (SLIT) in patients with allergic rhinoconjunctivitis have been assessed up to now in several trials [1–5], including Cochrane collaboration meta-analyses [6, 7]. Different methods, scores, and parameters have been developed to evaluate the efficacy, safety, and other properties of SLIT [8]. These tools include symptom, medication, and combined scores [9]. Furthermore, parameters such as the nasal provocation test, bronchial provocation tests, or titrated skin tests are also used to evaluate SLIT [9, 10].

Another important tool used to assess the efficacy of SLIT is the conjunctival provocation test (CPT). The CPT was developed decades ago [11] as a means to help diagnose allergic rhinoconjunctivitis and to evaluate the efficacy of an antiallergic therapy [12–14]. Möller et al. [15] rated the test as safe, easy, and precise. Since then, the validity and reproducibility of the CPT have been demonstrated in several investigations [16, 17], and its results have been shown to concord with those of nasal provocation tests [18].

Studies attempting to determine whether a correlation exists between preseasonal CPT findings and symptoms occurring during the following pollen season have reported heterogeneous results [19, 20]. Kruse et al. [19] concluded that preseasonal CPT results correlate with symptom severity and rescue medication use during the pollen season following a first course of preseasonal immunotherapy.

Based on the findings by Kruse et al., it would be desirable if clinical practice had a generally accepted parameter that is able to predict symptom severity even before a first course of preseasonal immunotherapy has been completed. Therefore, we performed a post hoc analysis of two prospective, randomized, double-blind, placebo-controlled trials to investigate whether the CPT can accomplish this task as early as 4 weeks after initiating SLIT.

## Methods

### Study populations

This post hoc analysis is based on two prospective, randomized, double-blind, placebo-controlled, multicenter, two-armed, phase III trials in participants with tree or grass pollen–related allergic rhinoconjunctivitis. The tree pollen trial included 22 sites, 18 of which enrolled participants in the study; the grass pollen trial included 23 sites, 15 of which randomized participants. Both trials were carried out in Germany with the approval of the Medical Ethics Committee of the North Rhine Medical Council (EudraCT numbers 2012-004916-79 (grass pollen trial) and 2013-002129-43 (tree pollen trial)) and the German Federal Ministry of Health (Paul-Ehrlich-Institut) [21, 22].

Adults aged 18–75 years with a history of at least 2 years of tree or grass pollen–induced allergic rhinitis and/or allergic rhinoconjunctivitis with or without seasonal, controlled allergic asthma were enrolled in the studies after having given written informed consent. Other inclusion criteria were: specific-IgE reactivity to tree or grass pollen (CAP-RAST ≥ class 2 (0.70 kU/L)), a positive screening skin prick test (SPT) (wheal diameter ≥ 3 mm, negative control < 2 mm), and a positive response to conjunctival provocation testing at both the screening and the inclusion visits. Participants with cosensitizations could be included if they did not suffer from typical symptoms caused by coseasonally prevalent allergens. Furthermore, specific IgE and SPT results for coseasonally prevalent allergens had to be lower than those for tree or grass pollen, respectively. The main exclusion criteria were: previous immunotherapy within 5 years prior to screening or ongoing immunotherapy for any allergen, predominant perennial allergic rhinitis, partly controlled or uncontrolled asthma, significant medical conditions, pregnancy, breastfeeding, lack of adequate contraception, and intake of contraindicated concomitant medication.

### Assignment and intervention

In all, 188 participants were randomized in the tree pollen study. During the treatment phase of at least 84 ± 14 days, participants received either LAIS^®^ birch–alder monomeric allergoid tablets at a daily dosage of 1000 units of allergen (UA) (manufactured by Lofarma S.p.A., Milan, Italy) or placebo tablets according to the randomization schedule.

A total of 90 participants were randomized in the grass pollen study. They received either LAIS^®^ grass tablets at a daily dosage of 1000 UA (Lofarma S.p.A., Milan, Italy) or placebo tablets for 20 weeks. Participants were instructed to place 1 sublingual tablet per day under the tongue and to let it dissolve for 2 min before swallowing. All participants were supplied with a blister package of an oral antihistamine (loratadine, 10 mg) as rescue medication for potentially appearing local side effects of the study medication.

### Randomization and blinding

Computer-generated randomization lists had a random block size of 4 in the tree pollen study and 8 in the grass pollen study. Participants were allocated to the next random treatment number in consecutive, ascending order. Blinding of the participants and the investigators was ensured by the identical shape, size, weight, color, taste, and smell of the study medication and its packaging. Labeled and sealed envelopes, one for each randomization code, contained the corresponding treatment allocation and were stored in a secure place at the respective study center. The envelopes were only to be opened in the case of a participant-related event requiring unblinding.

### Measurement and assessment

A CPT was performed at the screening visit (Visit 0), the inclusion visit before the first intake of study medication (Visit 1), after 4 weeks of treatment (Visit 3), and after 12 weeks of treatment in the grass pollen study (Visit 4) or at the end-of-study visit (calendar week 18–19) in the tree pollen study (Visit 5), respectively. ALK-lyophilized SQ provocation testing solutions (ALK-Abelló, Hørsholm, Denmark) in increasing concentrations containing 100, 1000, and 10,000 SQ/ml were applied to one of the participant’s eyes. Simultaneously, control solution was administered to the other eye to rule out false-positive reactions. This control procedure was carried out each time a new allergen concentration was tested. The investigator documented the participant’s conjunctival reaction 10 min after applying each concentration as described by Dogan et al. [23]. Reactions were graded according to Riechelmann et al. [18] (Table 1). Responses of stage II or higher were considered positive. The challenged eye was then rinsed with water and a topical antihistamine was applied. In case of a negative reaction, the next higher dosage was administered. If there was still no reaction to the highest allergen concentration, the test was rated as negative.

**Table 1:** Stages of the reactions to the conjunctival provocation test (CPT) according to Riechelmann et al. [18]

Stage	Findings
0	No subjective or visible reaction
I	Itching, reddening, foreign body sensation
II	Stage I + tearing, vasodilatation of conjunctiva bulbi
III	Stage II + vasodilatation and erythema of conjunctiva tarsi, blepharospasm
IV	Stage III + chemosis, lid swelling

**Fig. 1:**
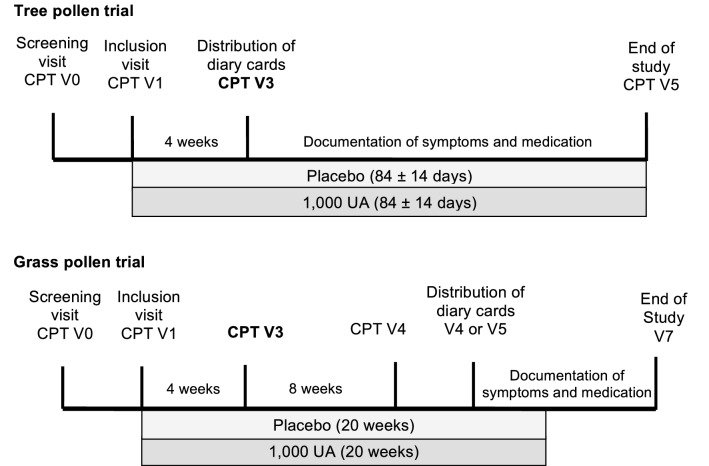
Study design. Timelines of study visits and procedures carried out in the two trials. *CPT* conjunctival provocation test, *UA* unit of allergen, *V* visit

Diaries were handed out to the participants after 4 weeks of treatment in the tree pollen study (Visit 3) and after 12–20 weeks of treatment in the grass pollen study (Visit 4 or Visit 5) (Fig. 1). Participants were asked to document the six rhinoconjunctivitis symptoms of sneezing, rhinorrhea, nasal pruritus, nasal congestion, ocular pruritus, and watery eyes on a daily basis for the respective pollen season. Each symptom was evaluated by the participant using the following score: 0 = absent symptoms; 1 = mild symptoms; 2 = moderate symptoms; 3 = severe symptoms (Table 2). The rhinoconjunctivitis symptom score (RTSS) was calculated by summing up the six individual symptom scores and could range from 0 to 18. The higher the score, the more severe the symptoms were in general.

**Table 2:** Definition of the symptom severity stages

Score	Severity
0	Absent symptoms: no sign/symptom evident
1	Mild symptoms: sign/symptom clearly present, but minimal awareness, easily tolerated
2	Moderate symptoms: definite awareness of sign/symptom that is bothersome, but tolerable
3	Severe symptoms: sign/symptom that is hard to tolerate; causes interference with the activities of daily living and/or with sleeping

Participants were also asked to record their daily intake of rescue medication. The total rescue medication score (TRMS) was calculated taking into account the use of oral antihistamines, levocabastine eye drops, and nasal corticosteroids according to the following escalation scheme: Step 1 = antihistamine (oral) 1 × 10 mg, maximal daily score of 6; Step 2 = Step 1 plus additional levocabastine (eyedrops) 2 × 1 drop per eye, maximal daily score of 9; Step 3 = Step 2 plus additional beclomethasone (nasal) 2 × 0.05 mg/nostril, maximal daily score of 18; only for asthmatic participants: long-acting b2-agonists 2 × 1–2 inhalations, 9 points in addition to the daily rescue medication score (Steps 1–3).

For each day of the assessment period, RTSS and TRMS were added together, yielding a daily total combined score (TCS) [24]. All individual daily TCS values were collected during the respective pollen period in order to calculate the mean daily scores within that period for RTSS, TRMS, and TCS for the peak of the tree pollen season, defined as those 14 consecutive days with “high” pollen concentrations (stage 3 according to the German Weather Service, Deutscher Wetterdienst [DWD]), for the peak 30 days of the tree/grass pollen season with a pollen count of at least “moderate” pollen concentrations (stage 2 according to the DWD), and for the entire tree/grass pollen season of 60 days, respectively [25].

The primary efficacy endpoint of the tree pollen study was the TCS for the 14-day peak of the birch pollen season (TCS 14), whereas the primary efficacy endpoint for the grass pollen study was defined as the 30-day peak of the grass pollen season (TCS 30). Secondary assessments for both studies were the remaining TCS, RTSS, and TRMS for the time periods mentioned above.

“Well days” were also determined, being defined as those days in the entire birch/grass pollen season having a maximum symptom score of 2 and no rescue medication use according to Durham et al. [5, 13].

Global evaluations of the therapies were carried out at the end of the studies. Participants were asked to evaluate the efficacy of their respective treatment, their satisfaction with the treatment, and whether they would recommend it to others based on a 4-point scale from 0 to 3, with 3 being the best rating.

### Statistical methods

The primary outcomes of these studies have been reported elsewhere [21, 22]. In the present post hoc analysis, actively treated participants (“SLIT participants”) were divided into two groups. The first group consisted of SLIT participants who had ceased to show a positive reaction (stage II or higher) to CPT after 4 weeks of treatment (“nonreactive group”). The second group consisted of all SLIT participants who still showed a reaction to the test after 4 weeks of treatment (“reactive group”). Reaction compared to baseline was tested using a Chi square test. The TCS, RTSS, TRMS, well days, and the global evaluation were defined as outcome parameters for each of the groups. A Mann–Whitney *U* test was performed to identify differences between the two groups with the level of significance set at 5%. The analysis of the data sets was performed using SPSS by IBM for Windows (Version 22, IBM Corp., Armonk, NY, USA).

## Results

### Study population

**Fig. 2:**
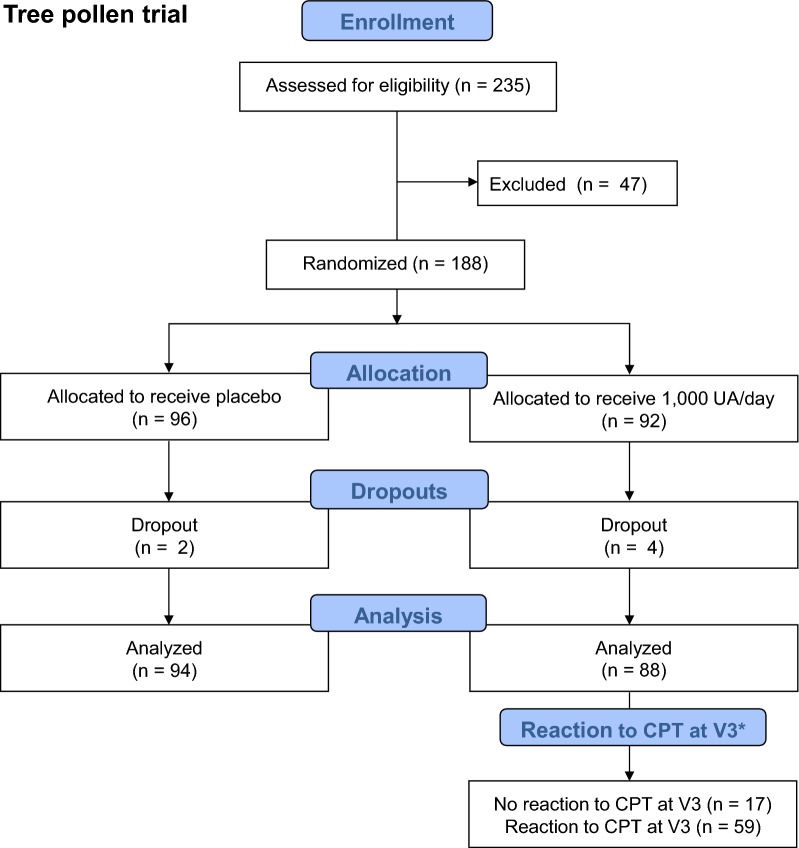
Flowchart of the tree pollen study population. *Due to an unforeseen shortage of CPT allergen solution on the manufacturer’s part, the CPT at V3 was optional, depending whether sufficient allergen solution was available. *CPT* conjunctival provocation test, *UA* unit of allergen, *V* visit

A total of 235 participants were screened for inclusion and exclusion criteria in the tree pollen study; 188 eligible participants were randomized (Fig. 2). Throughout the trial, 6 dropouts were recorded, leaving 182 evaluable participants at the end of the study. Of these 182 participants, 88 received active treatment. Due to an unforeseen shortage of CPT allergen solution on the manufacturer’s part, the CPT at Visit 3 was optional, depending whether sufficient allergen solution was available, leaving 76 participants eligible for the post hoc analysis.

**Fig. 3:**
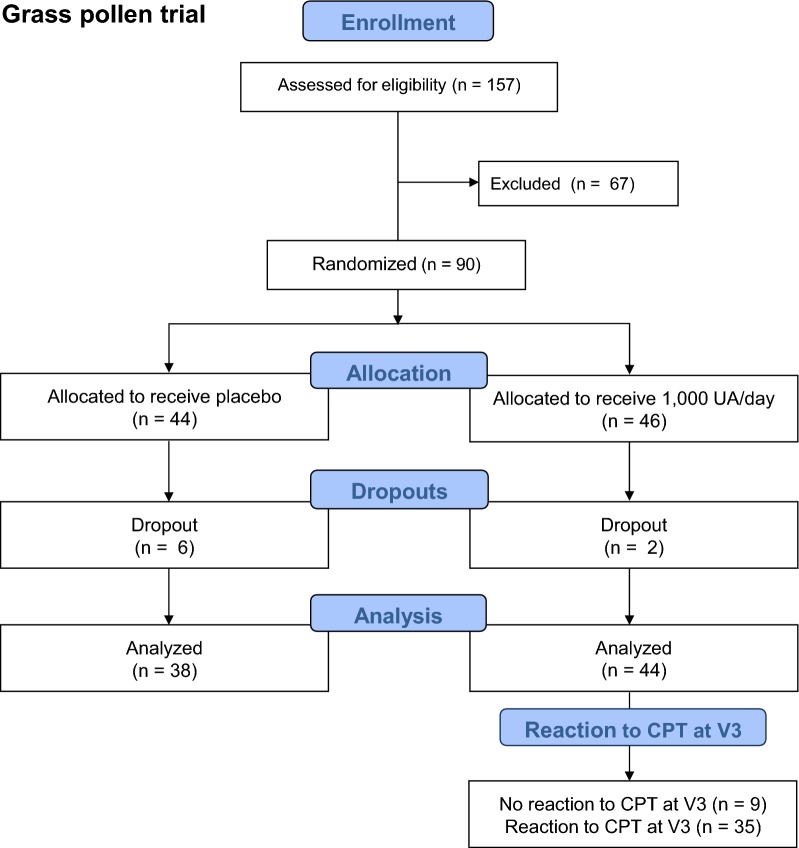
Flowchart of the grass pollen study population

In the grass pollen study, 157 participants were screened for inclusion and exclusion criteria. Of these, 90 eligible participants were randomized. Since there were 8 dropouts in the course of the study, a total of 82 participants were evaluable at the end of the grass pollen study, 44 of whom underwent active treatment (Fig. 3).

Demographic characteristics were similar between the two treatment groups within each study. Comparing participants who were nonreactive or reactive to CPT after 4 weeks of SLIT, demographic characteristics were homogeneous for both groups (Tables 3, 4). Concerning a positive response to conjunctival provocation testing at both screening and inclusion visit, the reactive and the nonreactive group were similar for that parameter (tree: *P* = 0.219; grass *P* = 0.668).

**Table 3:** Demographic characteristics of patients in the tree pollen study

	1000 UA/day		Placebo
	Reaction	No reaction	
*Patient population*			
n	59	17	94
*Male*			
n	26	10	34
%	44.1	58.8	36.2
*Female*			
n	33	7	60
%	55.9	41.2	63.8
*Mean age*			
yr	45.54	49.41	46.73
SD	13.30	13.86	11.96
*Mean duration of rhinitis*			
yr	21.32	22.29	20.63
SD	14.48	14.22	12.43
*Bronchial asthma*			
n	18	5	24
%	30.5	29.4	25.5
*Atopic dermatitis*			
n	0	0	1
%	0	0	1.1
*Birch pollen*			
n	All		
%	100		
*Alder pollen*			
n	57	17	89
%	98.3^a^	100	96.7^b^
*Grass pollen*			
n	31	12	57
%	52.5	70.6	60.6
*Rye pollen*			
n	30	12	49
%	50.8	70.6	52.1
*Hazel pollen*			
n	50	15	74
%	84.7	88.2	78.7
*House dust mite*			
n	21	3	25
%	35.6	17.6	26.6
*Cat dander*			
n	21	7	37
%	35.6	41.2	39.4
*Dog dander*			
n	16	4	30
%	27.1	23.5	31.9
*Alternaria*			
n	12	1	19
%	20.3	5.9	20.2

*SD* standard deviation, *UA* unit of allergen

^a^For alder pollen, SPT results from 1 patient are missing (n = 58)

^b^For alder pollen, SPT results from 2 patients are missing (n = 92)

**Table 4:** Demographic characteristics of patients in the grass pollen study

	1000 UA/day		Placebo
	Reaction	No reaction	
*Patient population*			
n	35	9	38
*Male*			
n	18	3	21
%	51.4	33.3	55.3
*Female*			
n	17	6	17
%	48.6	66.7	44.7
*Mean age*			
yr	39.31	40.11	37.63
SD	12.19	11.94	13.10
*Mean duration of rhinitis*			
yr	18.86	22.78	23.03
SD	12.94	13.07	12.54
*Bronchial asthma*			
n	2	0	4
%	5.7		10.5
*Atopic dermatitis*			
n	0	0	0
%	0	0	0
*Grass pollen*			
n	All		
%	100		
*Rye pollen*			
n	34	9	37
%	97.1	100	97.4
*Hazel pollen*			
n	9	2	11
%	25.7	22.2	28.9
*Ambrosia*			
n	4	0	6
%	11.4	0	15.8
*Birch pollen*			
n	11	2	16
%	31.4	22.2	42.1
*Mugwort*			
n	7	2	11
%	20.0	22.2	28.9
*House dust mite*			
n	4	2	8
%	11.4	22.2	21.1
*Cat dander*			
n	7	1	5
%	20.0	11.1	13.2
*Dog dander*			
n	7	3	6
%	20.0	33.3	15.8
*Alternaria*			
n	1	1	2
%	2.9	11.1	5.3

*SD* standard deviation, *UA* unit of allergen

### Pollen count

According to the DWD, the peak of the 2014 birch pollen season with a constant high pollen concentration of stage 3 on a scale from 0 to 3 (0 = 0, 1 = 1–10, 2 = 11–50, and 3 = 51 or more birch pollen grains per cubic meter) started on 28 March 2014 [25].

The peak of the 2014 grass pollen season with a constant pollen concentration of stage 2 (0 = 0, 1 = 1–5, 2 = 6–30, and 3 = 31 or more grass pollen grains per cubic meter) began on 21 or 22 May 2014 in all regions of Germany.

### Endpoints

#### Tree pollen study

For the post hoc analysis, SLIT participants (*n* = 76) in the tree pollen study were divided into nonreactive and reactive groups according to their CPT results after 4 weeks of treatment. Of these SLIT participants, 17 (22.4%) did not show a reaction to CPT after 4 weeks (*P* = 0.0001), whereas 59 participants (77.6%) did.

SLIT participants who did not show a reaction had a significantly lower combined score for the peak of the birch pollen season (TCS 14) (8.50) than those who still showed a reaction to CPT (*P* = 0.017). Likewise, the TCS for the 30-day peak (TCS 30) was significantly lower in the nonreactive group than in the reactive group (*P* = 0.042). The TCS for 60 days was also lower in the nonreactive group than in the reactive group but did not reach statistical significance (*P* = 0.104) (Table 5).

**Table 5:** Results of the tree pollen study

	Placebo					1000 UA				
	No reaction		Reaction		*P*	No reaction		Reaction		*P*
	Mean	SD	Mean	SD		Mean	SD	Mean	SD	
TCS 14	11.90	7.20	12.62	8.14	0.85	8.50	6.28	13.15	7.19	0.02
TCS 30	9.84	6.90	10.96	6.96	0.46	7.79	5.52	11.03	6.01	0.04
TCS 60	9.15	6.43	9.27	5.88	0.73	7.36	5.31	9.67	5.33	0.10
RTSS 14	7.10	3.11	6.96	4.16	0.75	4.64	2.78	6.72	3.47	0.02
RTSS 30	5.93	2.94	6.30	3.76	0.83	4.35	2.58	5.75	3.08	0.05
RTSS 60	5.55	2.54	5.49	3.03	0.90	3.94	2.40	5.17	2.66	0.07
TRMS 14	4.81	5.66	5.66	5.30	0.30	3.86	4.70	6.43	5.17	0.08
TRMS 30	3.91	5.16	4.66	4.52	0.26	3.44	4.19	5.29	4.38	0.10
TRMS 60	3.60	4.89	3.78	4.06	0.42	3.42	3.81	4.50	4.03	0.26
Well days	14.17	14.94	15.75	17.85	0.88	22.76	18.73	15.08	16.01	0.16
Efficacy	2.56	0.62	2.06	0.79	0.02	2.29	0.85	2.05	0.78	0.24
Satisfaction	2.11	0.90	1.97	0.85	0.49	2.18	0.81	1.92	1.01	0.42
Recommendation	2.17	0.79	2.33	0.86	0.32	2.41	0.71	2.15	0.93	0.36

*RTSS* rhinoconjunctivitis total symptom score, *SD* standard deviation, *TCS* total combined score, *TRMS* total rescue medication score, *UA* unit of allergen

Similarly, the RTSS 14 was significantly lower in SLIT participants who did not show a reaction to the CPT than that in participants who did (*P* = 0.024). Differences between the nonreactive and the reactive groups of SLIT participants could also be observed for the RTSS 30 (*P* = 0.053) and for the RTSS 60 (*P* = 0.065). However, these differences were not statistically significant (Table 5).

In terms of rescue medication, a trend towards less intake was observed in the TRMS 14, with a mean of 3.86 in SLIT participants without reactions and 6.43 in those with reactions (*P* = 0.082), as well as in the TRMS 30 for the group without reactions (3.44) in comparison to the group with reactions (5.29) (*P* = 0.103) (Table 5).

Also, when SLIT participants who ceased to show a reaction to the CPT after 4 weeks of treatment were compared with placebo-treated participants, there was a strong trend towards lower RTSS 14 (*P* = 0.061), RTSS 30 (*P* = 0.081), and RTSS 60 (*P* = 0.067) values for nonreactive SLIT participants (Table 5). For the placebo-treated patients, there were no significant differences between reactive and non-reactive patients except for the self-reported efficacy (*P* = 0.019).

#### Grass pollen study

Similar to the tree pollen study, SLIT participants (*n* = 44) in the grass pollen study were divided into nonreactive and reactive groups based on CPT results after 4 weeks of treatment. Of these SLIT participants, 9 (20.5%) did not react to the CPT after 4 weeks (*P* = 0.003) whereas 35 (79.5%) still did.

A numerical advantage was found which did not reach statistical significance due to the small sample size in the RTSS 30, with a mean of 3.30 in participants not showing a reaction and 5.29 in participants still reacting to CPT (*P* = 0.065), and in the RTSS 60, with a score of 2.84 in participants without reactions and 4.09 in those with reactions (*P* = 0.146). These results demonstrate a further trend towards fewer symptoms in the nonreactive group (Table 6).

**Table 6:** Results of the grass pollen study

	Placebo					1000 UA				
	No reaction		Reaction		*P*	No reaction		Reaction		*P*
	Mean	SD	Mean	SD		Mean	SD	Mean	SD	
TCS 30	12.68	5.69	9.81	6.79	0.39	7.13	4.88	9.68	7.41	0.37
TCS 60	7.63	3.27	7.31	5.32	0.69	6.08	4.34	7.54	6.60	0.74
RTSS 30	5.81	4.84	5.05	2.90	0.94	3.3	2.37	5.29	3.36	0.07
RTSS 60	3.64	2.82	3.85	2.30	0.69	2.84	1.95	4.09	2.95	0.15
TRMS 30	6.87	3.23	4.76	4.42	0.32	3.82	4.17	4.39	4.94	0.63
TRMS 60	3.98	2.18	3.47	3.59	0.50	3.24	3.79	3.45	4.20	0.64
Well days	29.67	5.51	27.11	17.01	0.81	28.22	18.32	25.14	17.65	0.57
Efficacy	2.33	0.58	1.91	0.78	0.36	2.67	0.50	2	0.77	0.02
Satisfaction	2.33	0.58	2.14	0.88	0.84	2.44	0.73	1.97	0.89	0.15
Recommendation	2.33	0.58	2.37	0.77	0.77	2.56	0.73	2.14	0.88	0.19

*RTSS* rhinoconjunctivitis total symptom score, *SD* standard deviation, *TCS* total combined score, *TRMS* total rescue medication score, *UA* unit of allergen

Furthermore, a significant difference (*P* = 0.019) could be observed with respect to the self-reported efficacy of the treatment between participants with reactions (2.00) and those without reactions (2.67), once again in favor of the nonreactive group (Table 6).

When comparing nonreactive SLIT participants with placebo-treated participants, a statistically significant difference with respect to the self-reported efficacy of the treatment (*P* = 0.010) could be observed as well as a trend towards a lower RTSS 30 (*P* = 0.086) in the nonreactive, actively treated group (Table 6). For the placebo-treated patients, there was no significant difference between reactive and non-reactive patients.

## Discussion

The findings from this study suggest that as soon as 4 weeks after initiation of SLIT, the CPT can reveal early responders who will profit from this therapy during the upcoming pollen season. This observation may prove valuable in the search for generally accepted biomarkers that can predict a patient’s response to allergen immunotherapy (AIT) [26].

In both trials of this post hoc analysis, nonreactivity in the CPT was observed in 22.4% (tree) and 20.5% (grass) of actively treated participants after 4 weeks of SLIT intake. Nonreactive SLIT participants in both trials showed trends towards lower symptom and rescue medication scores during the peak of the respective pollen season, more well days, as well as a better evaluation of efficacy, greater satisfaction, and more recommendations of SLIT treatment than did participants still reacting to conjunctival provocation. Similar trends could be observed when comparing nonreactive SLIT participants to placebo-treated participants.

Heterogeneous results have been reported with respect to a possible correlation between preseasonal CPT and rhinoconjunctivitis symptoms during the pollen season [19, 20]. Our findings support those presented by Kruse et al. [19], who showed that the CPT can be used effectively as a parameter for predicting allergic rhinoconjunctivitis symptoms during the pollen season after a first course of preseasonal immunotherapy. In a posttrial observation of two separate prospective, randomized, double-blind, controlled trials, the authors found significances when comparing groups with and without a positive response to the final conjunctival allergen challenge after 12 weeks of preseasonal immunotherapy with either birch–alder or grass pollen SLIT tablets. These significances became apparent when comparing the TCS and the use of rescue medication in both trials as well as in the RCS in the grass pollen trial and the number of well days in the tree pollen trial. Similar to Kruse et al., we also found significant differences during the pollen season in the TCS and the RTSS comparing patients reacting to CPT to those who did not. In contrast to Kruse et al., who performed the CPT after patients had completed the 12-week immunotherapy course and found that the CPT can be used as a predictive parameter for AR symptoms after immunotherapy, we performed the CPT as early as 4 weeks after initiating immunotherapy to investigate whether it can also be used as a predictive marker at such an early stage of ongoing immunotherapy.

On the contrary, Radcliffe et al. [20] were not able to show a correlation between preseasonal CPT results and seasonal symptoms, rescue medication use, or quality of life scores. They retrospectively analyzed 91 placebo-treated participants from a randomized, placebo-controlled study on low dosage AIT.

For SLIT, a treatment duration of approximately 3 years is usually recommended in order to achieve a therapeutic effect in patients with allergic rhinoconjunctivitis [27, 28]. Our results suggest that it is possible to predict the promising outcome of SLIT in an upcoming pollen season in about 20% of all actively treated patients based on the patient’s nonreaction to the CPT after only 4 weeks of treatment. Thus, patients who are likely to benefit from ongoing therapy can be detected at an early stage of therapy. One has to concede that to date there has been no harmonization as to what can be considered a clinically significant beneficial outcome [29].

Horak et al. [30] aimed to demonstrate such an early response to SLIT. In a double-blind, placebo-controlled trial, they found significant improvement in rhinoconjunctivitis symptoms beginning from the first month of treatment with grass pollen SLIT tablets. Their trial was not performed during the pollen season but under controlled conditions in an allergen challenge chamber without rescue medication. The RTSS was assessed every 15 min during a 4-h allergen exposure challenge at baseline as well as after 1 week, 1, 2, and 4 months. A significant treatment effect was detectable after the first month of treatment (*P* = 0.0042), which could be maintained at 2 (*P* = 0.0203) and 4 months (*P* = 0.0007) with decreasing average RTSS (ARTSS) results at each challenge in the SLIT group. In contrast to Horak et al., we performed a CPT instead of provocation in an allergen challenge chamber, but even so, we found a similar early onset of nonreactivity in the CPT after only 4 weeks of SLIT intake. In the subsequent pollen season, the anticipated beneficial outcome was confirmed.

Early onset of an immunotherapy effect was also found in another trial conducted by Ott et al. [3]. In a randomized, double-blind, placebo-controlled, parallel-group, multicenter trial, the authors were able to demonstrate the efficacy of coseasonal grass pollen SLIT from the first season onwards. Compared to baseline values, the combined score (including symptoms and medication) decreased significantly more in participants undergoing SLIT than in those receiving placebo, already during the first season of SLIT intake. Since SLIT was only administered coseasonally and a treatment effect could already be observed within the same season, one may assume an early response to SLIT similar to the one we observed.

There were also placebo-treated patients who did not react to conjunctival challenge after 4 weeks of treatment. This effect was already described by Gloistein et al. [31]. They found that repeated conjunctival provocation may have a hyposensitizing effect. In contrast to the actively treated patients, there was no significant difference in symptoms and medication usage in the pollen season for the placebo-treated patients between reactive and non-reactive patients. The placebo-treated patients lacked the daily sublingual confrontation with the allergen, but actively treated patients became symptom-free upon natural pollen exposure during the pollen season. This shows that CPT has a positive predictive value regarding the clinical outcome of SLIT. Considering these results, one may assume that the CPT can reveal patients who are receptive for hyposensitizing effects and who will therefore profit from ongoing sublingual immunotherapy.

One strength of the analysis presented here is that two prospective, double-blind, randomized, placebo-controlled, multicenter, phase III studies yielded similar results demonstrating a similar trend—independently of each other. Furthermore, in contrast to Kruse et al., who performed a posttrial observation, our analysis was carried out based on data gathered according to the study protocol, and no further data had to be collected to complete the analysis.

An important limitation, on the other hand, is the fact that we conducted a post hoc analysis. By doing so, more value may be ascribed to the outcome of an unplanned analysis than is warranted [32]. Moreover, the grass pollen trial did not reach the calculated sample size due to recruiting difficulties, and the CPT on V3 could not be performed in all patients due to a shortage of allergen test solution. Because of this, further investigations seem necessary to confirm the trends observed in our analysis.

## Conclusion

Our data suggest that the cease in SLIT patients’ reactions to preseasonal CPT reflects their individual outcomes during the subsequent pollen season. Carrying out the CPT after only 4 weeks of SLIT can reveal early responders to therapy. Thus, a prediction concerning treatment efficacy and possible symptoms during an upcoming pollen season can already be made at an early stage of treatment with SLIT. This estimation could help increase compliance in patients receiving preseasonal SLIT and help physicians improve their patients’ individual therapeutic strategies for the upcoming pollen season by knowing whether to prescribe rescue medication as needed or on a daily basis.

## Abbreviations

AIT: allergen immunotherapy
CAP-RAST: carrier polymer system-radioallergosorbent test
CPT: conjunctival provocation test
DWD: Deutscher Wetterdienst (German Weather Service)
RTSS: rhinoconjunctivitis total symptom score
SLIT: sublingual immunotherapy
SPT: skin prick test
TCS: total combined score
TRMS: total rescue medication score
UA: unit of allergen

## References

[1] Didier A, Malling HJ, Worm M, Horak F, Jäger S, Montagut A (2007). Optimal dose, efficacy, and safety of once-daily sublingual immunotherapy with a 5-grass pollen tablet for seasonal allergic rhinitis. J Allergy Clin Immunol.

[2] Khinchi MS, Poulsen LK, Carat F, André C, Hansen AB, Malling HJ (2004). Clinical efficacy of sublingual and subcutaneous birch pollen allergen-specific immunotherapy: a randomized, placebo-controlled, double-blind, double-dummy study. Allergy.

[3] Ott H, Sieber J, Brehler R, Fölster-Holst R, Kapp A, Klimek L, et al. Efficacy of grass pollen sublingual immunotherapy for three consecutive seasons and after cessation of treatment: the ECRIT study. Allergy. 2009;64(1):179–86. Erratum in Allergy. 2009;64(9):1394–401. 10.1111/j.1398-9995.2009.02194.x. 10.1111/j.1398-9995.2008.01875.x 19076534

[4] Pfaar O, van Twuijver E, Boot JD, Opstelten DJ, Klimek L, van Ree R (2016). A randomized DBPC trial to determine the optimal effective and safe dose of a SLIT-birch pollen extract for the treatment of allergic rhinitis: results of a phase II study. Allergy.

[5] Durham SR, Emminger W, Kapp A, de Monchy JG, Rak S, Scadding GK (2012). SQ-standardized sublingual grass immunotherapy: confirmation of disease modification 2 years after 3 years of treatment in a randomized trial. J Allergy Clin Immunol.

[6] Radulovic S, Wilson D, Calderon M, Durham S (2011). Systematic reviews of sublingual immunotherapy (SLIT). Allergy.

[7] Calderon MA, Penagos M, Sheikh A, Canonica GW, Durham SR (2011). Sublingual immunotherapy for allergic conjunctivitis: Cochrane systematic review and meta-analysis. Clin Exp Allergy.

[8] European Medicines Agency. Guideline on the Clinical Development of Medicinal Products for the Treatment of Allergic Rhinoconjunctivitis. London, 21 October 2004. Document CHMP/EWP/2455/02. http://www.ema.europa.eu/docs/en_GB/document_library/Scientific_guideline/2009/09/WC500003554.pdf. Accessed 9 Dec 2016.

[9] Pfaar O, Kleine-Tebbe J, Hörmann K, Klimek L (2011). Allergen-specific immunotherapy: which outcome measures are useful in monitoring clinical trials?. Immunol Allergy Clin N Am.

[10] Calderon MA, Eichel A, Makatsori M, Pfaar O (2012). Comparability of subcutaneous and sublingual immunotherapy outcomes in allergic rhinitis clinical trials. Curr Opin Allergy Clin Immunol.

[11] Peshkin MM (1931). XI. A dry pollen ophthalmic test in pollen asthma and hay fever patients negative to cutaneous tests. J Allergy.

[12] Friedlaender MH (2002). Conjunctival provocation testing: overview of recent clinical trials in ocular allergy. Curr Opin Allergy Clin Immunol.

[13] Durham SR, Yang WH, Pedersen MR, Johansen N, Rak S (2006). Sublingual immunotherapy with once-daily grass allergen tablets: a randomized controlled trial in seasonal allergic rhinoconjunctivitis. J Allergy Clin Imunol.

[14] Melillo G, Bonini S, Cocco G, Davies RJ, de Monchy JG, Frølund L (1997). EAACI provocation tests with allergens. Report prepared by the European Academy of Allergology and Clinical Immunology Subcommittee on provocation tests with allergens. Allergy.

[15] Möller C, Björksten B, Nilsson G, Dreborg S (1984). The precision of the conjunctival provocation test. Allergy.

[16] Abelson MB, Chambers WA, Smith LM (1990). Conjunctival allergen challenge. A clinical approach to studying allergic conjunctivitis. Arch Ophthalmol.

[17] Rimås M, Gustafsson PM, Kjellman N-IM, Björkstén B (1992). Conjunctival provocation test: high clinical reproducibility but little local temperature change. Allergy.

[18] Riechelmann H, Epple B, Gropper G (2003). Comparison of conjunctival and nasal provocation test in allergic rhinitis to house dust mite. Int Arch Allergy Immunol.

[19] Kruse K, Gerwin E, Eichel A, Shah-Hosseini K, Mösges R (2015). Conjunctival provocation tests: a predictive factor for patients’ seasonal allergic rhinoconjunctivitis symptoms. J Allergy Clin Immunol Pract.

[20] Radcliffe MJ, Lewith GT, Prescott P, Church MK, Holgate ST (2006). Do skin prick and conjunctival provocation tests predict symptom severity in seasonal allergic rhinoconjunctivitis?. Clin Exp Allergy.

[21] Efficacy and safety of sublingual immunotherapy with Allergoid LAIS^®^ Birch-Alder tablets for patients with tree pollen-induced allergic rhinoconjunctivitis, a Phase III study—ICH clinical study report. https://portal.dimdi.de/data/ctr/O-1943_01-2-0-E55F50-20150708100309.pdf. Accessed 9 Dec 2016.

[22] Efficacy and safety of sublingual immunotherapy wit Allergoid LAIS^®^ Grass tablets for patients with grass pollen-induced allergic rhinoconjunctivitis, a Phase III study—ICH clinical study report. https://portal.dimdi.de/data/ctr/O-1767_04-2-0-861038-20150909091138.pdf. Accessed 9 Dec 2016.

[23] Dogan S, Astvatsatourov A, Deserno TM, Bock F, Shah-Hosseini K, Michels A (2014). Objectifying the conjunctival provocation test: photography-based rating and digital analysis. Int Arch Allergy Immunol.

[24] European Medicines Agency. Guideline on the clinical development of products for specific immunotherapy for the treatment of allergic diseases. London, 20 November 2008. Document CHMP/EWP/18504/2. http://www.ema.europa.eu/docs/en_GB/document_library/Scientific_guideline/2009/09/WC500003605.pdf. Accessed 29 Nov 2016.

[25] Pollenflug-Gefahrenindex: Deutscher Wetterdienst. http://www.dwd.de/DE/leistungen/gefahrenindizespollen/gefahrenindexpollen.html. Accessed 01 Jan 2017.

[26] Shamji MH (2014). Mechanisms of allergen-specific sublingual immunotherapy and the use of biological markers in allergic rhinitis. Curr Treat Options Allergy.

[27] Larsson O, Hellkvist L, Peterson-Westin U, Cardell LO (2016). Novel strategies for the treatment of grass pollen-induced allergic rhinitis. Expert Opin Biol Ther.

[28] Jutel M, Agache I, Bonini S, Burks AW, Calderon M, Canonica W (2015). International consensus on allergy immunotherapy. J Allergy Clin Immunol.

[29] Nelson HS, Calderon MA, Bernstein DI, Casale TB, Durham SR, Andersen JS (2017). Allergen immunotherapy clinical trial outcomes and design: working toward harmonization of methods and principles. Curr Allergy Asthma Rep.

[30] Horak F, Zieglmayer P, Zieglmayer R, Lemell P, Devillier P, Montagut A (2009). Early onset of action of a 5-grass-pollen 300-IR sublingual immunotherapy tablet evaluated in an allergen challenge chamber. J Allergy Clin Immunol.

[31] Gloistein C, Astvatsatourov A, Allekotte S, Mösges R (2015). Digitally analyzed conjunctival redness: does repeated conjunctival provocation intrinsically cause local desensitization of the eye?. Int Arch Allergy Immunol.

[32] Curran-Everett D, Milgrom H (2013). Post-hoc data analysis: benefits and limitations. Curr Opin Allergy Clin Immunol.

